# Reference materials and representative test materials to develop nanoparticle characterization methods: the NanoChOp project case

**DOI:** 10.3389/fchem.2015.00056

**Published:** 2015-10-19

**Authors:** Gert Roebben, Vikram Kestens, Zoltan Varga, Jean Charoud-Got, Yannic Ramaye, Christian Gollwitzer, Dorota Bartczak, Daniel Geißler, James Noble, Stephane Mazoua, Nele Meeus, Philippe Corbisier, Marcell Palmai, Judith Mihály, Michael Krumrey, Julie Davies, Ute Resch-Genger, Neelam Kumarswami, Caterina Minelli, Aneta Sikora, Heidi Goenaga-Infante

**Affiliations:** ^1^Institute for Reference Materials and Measurements, Joint Research Centre, European CommissionGeel, Belgium; ^2^Institute of Materials and Environmental Chemistry, Research Centre for Natural Sciences, Hungarian Academy of SciencesBudapest, Hungary; ^3^Physikalisch-Technische BundesanstaltBerlin, Germany; ^4^LGC LimitedTeddington, UK; ^5^Biophotonics Division 1.10, Federal Institute for Materials Research and TestingBerlin, Germany; ^6^Analytical Sciences, National Physical LaboratoryTeddington, UK

**Keywords:** nanoparticle, materials characterization, reference material, analytical quality assurance, metrology

## Abstract

This paper describes the production and characteristics of the nanoparticle test materials prepared for common use in the collaborative research project NanoChOp (Chemical and optical characterization of nanomaterials in biological systems), *in casu* suspensions of silica nanoparticles and CdSe/CdS/ZnS quantum dots (QDs). This paper is the first to illustrate how to assess whether nanoparticle test materials meet the requirements of a “reference material” (ISO Guide 30, 2015) or rather those of the recently defined category of “representative test material (RTM)” (ISO/TS 16195, 2013). The NanoChOp test materials were investigated with small-angle X-ray scattering (SAXS), dynamic light scattering (DLS), and centrifugal liquid sedimentation (CLS) to establish whether they complied with the required monomodal particle size distribution. The presence of impurities, aggregates, agglomerates, and viable microorganisms in the suspensions was investigated with DLS, CLS, optical and electron microscopy and via plating on nutrient agar. Suitability of surface functionalization was investigated with attenuated total reflection Fourier transform infrared spectrometry (ATR-FTIR) and via the capacity of the nanoparticles to be fluorescently labeled or to bind antibodies. Between-unit homogeneity and stability were investigated in terms of particle size and zeta potential. This paper shows that only based on the outcome of a detailed characterization process one can raise the status of a test material to RTM or reference material, and how this status depends on its intended use.

## Introduction

Most nanoparticles [particles with all external dimensions smaller than 100 nm (ISO/TS 27687, 2008)] are of natural origin or are incidental by-products of human activities, such as engine exhaust particles. Other nanoparticles are produced on purpose to have unique properties and to improve the performance of consumer products. The use of these manufactured nanoparticles leads to their occurrence in the environment and potential exposure of the human body to nanoparticles. Moreover, some nanoparticles are developed for intentional administration to the human body for biomedical applications (Minelli et al., 2010; Natte et al., 2012; Holzinger et al., 2014). Therefore, the potential health effects of nanoparticles are a cause for concern, and the number of studies to evaluate the toxicity of nanomaterials is increasing. Several authors have questioned the poor reproducibility of data from nanotoxicity studies (Warheit, 2010; Nature Nanotechnology, 2012; Ratna and Shard, 2013; Krug, 2014). One remediating measure is increased interlaboratory collaboration, which is actively supported by, e.g., EU authorities and funding agencies.

Collaborative research projects often set up interlaboratory comparisons to demonstrate the reproducibility of measurement methods, or, at least, to develop an understanding of their lack of reproducibility, based on the comparison of results from different laboratories obtained on similar test samples from a single batch or a common source. Unfortunately, it is often debated whether the observed differences are due to the method (which can be inherently difficult to reproduce) or due to differences in how the tests are performed in different laboratories or whether they are simply caused by differences between the samples tested in different laboratories. In order to prevent such debates, it is recommended to test a representative selection of test samples in one laboratory to confirm their homogeneity (i.e., properties of different samples would not differ from each other beyond a specified acceptable level) and their stability (i.e., properties of different samples would not change during transport or storage beyond a specified accepted level). Such demonstrated sufficient homogeneity and stability for a defined use are the main characteristics of a reference material (ISO Guide 30, 2015).

Generic guidance on the production and use of reference materials (RMs) is documented in a set of ISO Guides (ISO Guide 35, 2006; ISO Guide 34, 2009; ISO DGuide 31, 2014; ISO Guide 80, 2014; ISO Guide 33, 2015), which also explain the difference between RMs and certified reference materials (CRMs): CRMs are not only sufficiently homogeneous and stable for a specified intended use (as are all RMs), they also have been characterized in a thorough manner that allowed the RM producer to certify one or more values of the material properties, including their metrological traceability and an estimate of their uncertainty. This certification process requires considerable effort and investment. More importantly, in emerging measurement fields, such as in the measurement of material properties at the nanoscale, the traceability and uncertainty requirements imply that the development of CRMs is often not (yet) possible. This explains why the number of nanoparticle CRMs is still very limited (e.g., Braun et al., 2012; Stefaniak et al., 2013).

The production of RMs crucially depends on an accurate description of their intended use: without referring to the purpose it is supposed to serve, it is not possible to assess whether an RM is *sufficiently* homogeneous or stable. As a result, an RM is only for a specific, defined purpose. For other properties the RM can be a representative test material (RTM): “material, which is sufficiently homogenous and stable with respect to one or more specified properties, and is *implicitly* assumed to be fit for its intended use in the development of measurement and test methods that target properties other than those for which homogeneity and stability have been demonstrated.” This term was recently proposed (Roebben et al., 2013) and defined in ISO/TC 229 “Nanotechnologies” (ISO/TS 16195, 2013). Nanotechnology is one of the emerging measurement areas in which RTMs are effectively used.

An important question in the preparation of a collaborative project is which materials will be tested. RMs suitable for the specific aims of a project are often not available. Instead, a common set of test materials needs to be sourced and processed, and characterized to check whether they meet the combined technical needs of the project within the constraints (financial and time resources) of the project. This is an often underestimated aspect of collaborative research projects.

The authors of this paper were partners in NanoChOp (Chemical and optical characterization of nanomaterials in biological systems), a collaborative research project in the European Metrology Research Programme (EMRP), which addressed two analytical challenges. The first challenge was the detection and quantification of nanoparticles in biological media, and the analysis of their size (equivalent diameters) and surface charge (zeta potential) in the same media. The second ambition was to measure the optical properties (e.g., luminescence quantum yield) of fluorescently labeled or stained nanoparticles in such media. This paper provides the details of the production and properties of the main NanoChOp test materials, based on which other publications have been prepared (e.g., Bartczak et al., 2015) or are being prepared. At the same time, the paper provides a first and detailed illustration of the assessment of the RM or RTM status of nanoparticle test suspensions.

## Materials and methods

### Selection of test materials and targeted property values based on the intended use

Most existing nanoparticle CRMs are developed to calibrate particle size analysis (PSA) instruments. This requires a clear metrological traceability and a low uncertainty of the certified size values, which currently can only be obtained for spherical particles with monomodal nanoparticle size distributions. However, it has been pointed out (e.g., by Orts-Gil et al., 2013) that the “monomodal” (C)RMs are hardly representative for the nanomaterials produced industrially in large volumes. Therefore, while they are suitable as calibrants, they may not be very useful to assess the method performance for polydisperse and non-spherical nanoparticles.

It is important to recognize the different purposes of experimental studies (Krug, 2014). On the one hand, experiments can be performed to elucidate mechanistic differences in the behavior of (nano-)materials. This perspective encourages the scientist to select specific materials and test conditions which can reveal correlations between the behavior of the test materials and their physicochemical characteristics (size, shape, composition …). On the other hand, in a regulatory context, many experiments are conducted to assess the behavior of a particular industrially relevant material, which is often chemically not very pure and/or polydisperse. Krug and others (Crist et al., 2013) advocate for a more detailed physicochemical characterization of these complex materials. This requires methods that can deal with polydispersity and the corresponding quality control tools (e.g., certified multimodal nanoparticle size RMs, Kestens and Roebben, 2014).

Research projects often intend to make progress both in terms of development of methods suitable for more complex materials and test conditions, and in terms of mechanistic understanding of the behavior of the tested materials. In the case of NanoChOp, the challenge of measuring broad size distributions in complex biological media (for details: http://nanochop.lgcgroup.com/), would have produced a high level of complexity in the interpretation of the results. It is possible to detect nanoparticles and measure their (equivalent) diameters in suspensions under certain conditions (colloidal stability, concentration range matching the working range of the methods, etc.), but this is much more challenging if the particles occur in media relevant to toxicology (Orts-Gil et al., 2011). NanoChOp therefore decided to exclude a number of complicating material parameters, namely shape (Gallego-Urrea et al., 2014), polydispersity and aggregation state, and in general the presence of impurities that can interact with the biologically relevant systems. Hence it was decided to work with clean, aqueous suspensions of near-spherical nanoparticles with monomodal particle size distribution. A substantial part of the NanoChOp project focused on optical properties of nanoparticles, requiring a set of fluorescently labeled nanoparticle materials. Also, one material would serve as a model nanoparticle with optical parameters relevant for use in fluorescence-based immunoassays. The defined size and polydispersity would help to understand the influence of these factors on assay response. The bioassay format addressed was a typical rapid point-of-care immunoassay (Worsley et al., 2012), which requires the attachment of antibodies to the surface of the nanoparticle in such a way that they retain their immuno-reactivity.

It was therefore decided to search for three suspensions of application-relevant nanoparticles, ideally one of silica nanoparticles, one of titanium dioxide nanoparticles, and one of nanoparticles with particular luminescence properties, such as quantum dots (QDs). The selected silica material would be provided in aminated form, ready for labeling with different fluorophores using conventional NHS ester chemistry to enable direct comparison of the results of fluorescently labeled and non-labeled materials. Table 1 summarizes the specific target material parameters which had to be met in addition to more general requirements (spherical particles, aqueous suspensions, neutral pH, free of impurities, aggregates and agglomerates and of particles larger than 500 nm).

**Table 1:** **Initial target material characteristics and property values**.

**Material properties**	**Target values/characteristics**		
	**SiO_2_**	**TiO_2_**	**Quantum dots**
Size (nominal particle diameter)	50 nm	20 nm	30 nm
Polydispersity (FWHM/mean diameter)	<0.25	<0.25	<0.25
Surface functionalization	Aminated	None	Aminated or carboxylated
Zeta potential (absolute value)	>10 mV	>10 mV	>30 mV
Mass fraction	>5 g/kg	>5 g/kg	About 20 g/kg
Amount (mass of particles)	12 g	7 g	7 g
Shelf-life (in closed containers)	18 months	18 months	18 months
Shelf-life (after opening containers)	5 days	5 days	5 days

Initially, it was also considered to provide the materials as dispersions in a diluted fetal bovine serum. There is evidence that dispersions of nanoparticles in complex matrices can meet the homogeneity and stability criteria of a RM (e.g., Grombe et al., 2014). However, since the electrostatic repulsion that provides colloidal stability would be eliminated by the high ionic strength of the serum solutions, suspensions of nanoparticles in biological media suffer from stability problems when stored for too long or transported under non-ideal conditions (see e.g., Nabiul Afrooz et al., 2014). Also, it is known that in these complex matrices, nanoparticles develop a biomolecular corona (Monopoli et al., 2012), with an equilibrium composition that is difficult to predict, even if it can be monitored with modern spectrometric techniques (Docter et al., 2014). Therefore, instead of preparing all serum dispersions centrally and at once, protocols for dispersing the nanoparticle suspensions in serum were developed for use in the partner laboratories.

### Sourcing and processing of suitable nanoparticle materials

Table 1 also indicates the amount of material required. The values shown were calculated as the sum of the amounts of each material needed by the project partners in the different work packages of the NanoChOp project. An important parameter in this calculation was the sample intake of the techniques planned to be developed or validated in this measurement-method focused project. As the required amount of each material for the 3 year and multi-partner project was significant, and since the differences between batches of nanoparticle materials produced on a lab-scale typically do not match the targeted low polydispersity (Table 1), it was preferred to procure base materials from commercial suppliers. The issue of polydispersity has dominated discussions between material suppliers and NanoChOp project partners for a long time because the term polydispersity has multiple interpretations. It was agreed to use the following definition of polydispersity, based on the full width at half of the maximum height (FWHM) of the (main) mode in the size distribution:

$$\begin{array}{l} polydispersity=\frac{\begin{array}{c} FWHM\;of\;the\;peak\;in\;the\;particle\;size\\ distribution \end{array}}{\begin{array}{c} average\;equivalent\;diameter\;of\;the\;peak\;in\;the\\ particle\;size\;distribution \end{array}} \end{array}$$

Although this polydispersity value depends on the method used to measure the size distribution, a single maximum polydispersity of 0.25 was specified for the NanoChOp test materials (See Table 1).

Table 2 summarizes basic data about the source and further processing of the 6 nanoparticle test materials (NanoChOp-01 to -06) for which results will be shown in this paper. More details of the processing of the three finally selected materials (NanoChOp-03, -05, and -06) are provided here, as these are the materials based on which a number of other publications are being prepared by NanoChOp project partners. Details of the other three materials are provided in Supplementary Information (1).

**Table 2:** **Principal processing parameters of the NanoChOp suspensions**.

	**NanoChOp-01**	**NanoChOp-02**	**NanoChOp-03**	**NanoChOp-04**	**NanoChOp-05**	**NanoChOp-06**
Core particles	Silica (with organic fraction)	Silica	CdSe/CdS/ZnS	Silica (with organic fraction)	Silica	Silica
Surface groups	-NH_2_	None	Amine—PEG	-COOH	None	-NH_2_
Base material (supplier)	10 g/kg (microParticles, Berlin, DE)	Research grade, 20 g/kg (Grace, Columbia, MD, USA)	CANdot Series A CSS, 5 mM (~20 g/kg) (CAN, Hamburg, DE)	10 g/kg (microParticles, DE)	300 g/kg, Klebosol 30R50 (AZ Electronic Materials, Trosly Breuil, FR)	300 g/kg, Klebosol 30R50 (AZ Electronic Materials, Trosly Breuil, FR)
Prevention of microbiological contamination	Regular RM processing practice	Ampouling in mobile clean cell	Regular RM processing practice	–	Regular RM processing practice	Amination and ampouling in (mobile) clean cell
Rinsing volume	50 mL	150 mL	50 mL	–	>150 mL	50 mL
Particle concentration or mass fraction in ampoule	2.5 g/kg	2.5 g/kg	1 mM (~4 g/kg)	–	2.5 g/kg	2.5 g/kg
Amount of suspension per ampoule (ampoule volume)	2.5 mL (in 5 mL)	2.5 mL (in 5 mL)	2.5 mL (in 5 mL)	–	9 mL (in 10 mL)	1.9–2 mL (in 2 mL)
Number of ampoules	523	540	228	–	400 (taken from batch of 1871)	536
Storage temperature after processing	4°C	4°C	4°C	–	18°C	4°C
Remediation of microbiological contamination	Gamma irradiation (6 kGy, part of batch)	Autoclaving (part of batch)	Gamma irradiation (6 kGy)	–	Long term storage in air-tight closed glass ampoules	Not required

#### NanoChOp-03: Quantum dots

NanoChOp-03 was prepared from an aqueous suspension of CdSe/CdS/ZnS QDs covered with a shell of PEG molecules with amine end-functions (CANdots® Series A aqua, catalog number A4161002, Lot number SAL-0-182, CAN GmbH, Hamburg, DE). For cost reasons the initially foreseen amount of particles (Table 1) was significantly reduced. The base suspension was diluted with ultrapure water (reverse osmosis purified and sanitized) to the desired nominal concentration and filled in pre-scored amber glass ampoules (NAFVSM, Nijmegen, NL), following processing parameters summarized in Table 2. Immediately after filling, the ampoules were flushed with Ar and flame-sealed on an automatic ampoule filling machine (ROTA Verpackungstechnik, Wehr, Germany).

Absorption and emission spectra of the QDs were measured and confirmed the values given by the supplier (**Table 6**). After reports of viable microorganisms in NanoChOp-03, and having compared several sterilization options (see Section Remediation and Sterilization) the NanoChOp-03 ampoules were gamma irradiated with a ^60^Co based GS6000 pallet irradiator at Synergy Health (Etten-Leur, NL), with target doses between 5 and 10 kGy.

#### NanoChOp-05: Non-functionalized colloidal silica

**Figure 1:**
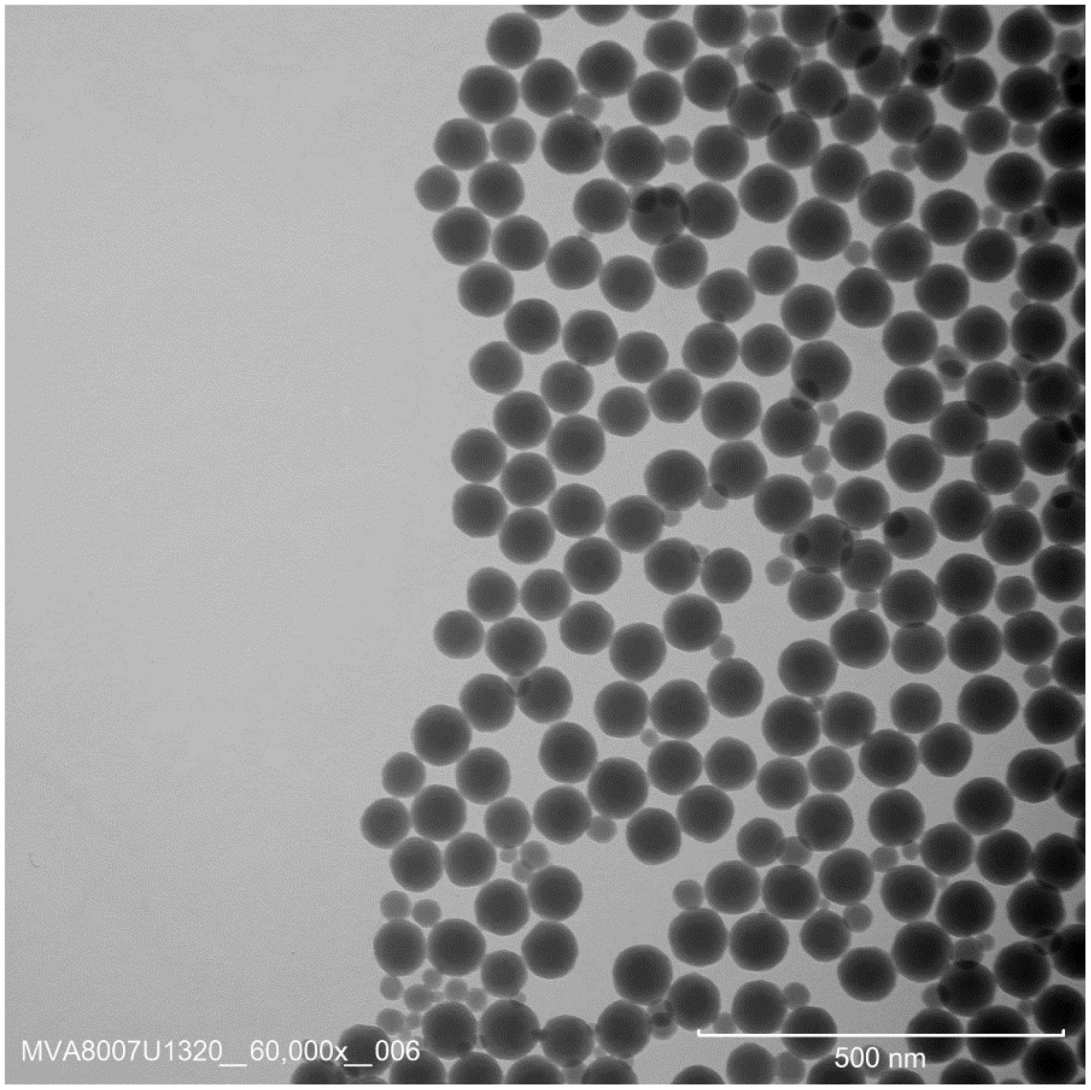
**Representative TEM image of the Klebosol 30R50 colloidal silica, from which NanoChOp-05 and NanoChOp-06 were prepared**.

Colloidal silica samples from several suppliers were compared in terms of polydispersity using DLS, CLS, and SAXS. A previously ampouled silica material, with proven absence of a viable microbiological load was selected to become NanoChOp-05. This material was prepared from Klebosol 30R50 (AZ Electronic Materials, Trosly, Breuil, FR), a 300 g/kg aqueous sol of non-porous silica particles grown in a liquid-phase process, which was diluted and ampouled generally in the same way as NanoChOp-03 (Table 2). Figure 1 shows a TEM image of the dense, spherical silica particles in the Klebosol 30R50 starting material.

#### NanoChOp-06: Aminated colloidal silica

The NanoChOp-06 base material was prepared from the batch of Klebosol 30R50 used to produce NanoChOp-05, following a previously described amination protocol (Pálmai et al., 2013). The base suspension was filtered using a Whatman cellulose filter (pore size range of 4–7 μm). The resulting loss of particles was limited, but not quantified. The filtered suspension was diluted with ethanol and dialysed for 3 days against ethanol (a.r., 99.98%, max. 0.02% water, Reanal) using a cellulose membrane tubing (Sigma-Aldrich, Ø76 mm, NMWL 12,400). The concentration of the particles in ethanol was 7.5 g/L. The amination reaction was carried out at 60°C for 15 min using 3-aminopropyl-diethoxy-methylsilane (APDEMS, 97%, Sigma-Aldrich) in an excess amount, and stopped by the addition of glacial acetic acid (EMSURE, Merck). The suspension was dialysed against autoclaved ultrapure water containing acetic acid (ca. 0.0016 L/L, pH 3) for 4 days under sterile conditions resulting in an aqueous suspension with particle mass fraction of approximately 4.6 g/kg. Part of the suspension was diluted to obtain an amount of 1.25 L with a particle mass fraction of 2.5 g/kg and pH 3, to prepare NanoChOp-06. The rest of the non-diluted was used for the production of fluorophore-labeled particles.

To avoid contamination of the sterile NanoChOp-06 base material during ampouling, the procedure used for NanoChOp-03 was modified. Prior to filling the glass ampoules, they were opened and heated in an oven for >2 h to 130°C for sterilization [see also Supplementary Information (2)]. The ampoule filling installation was placed in a mobile clean-cell (Terra Universal, Romex, Rhenen, NL) in which air filtered with a high-efficiency particulate air filter was recirculated to achieve an ISO 6 air cleanliness level (ISO 14644-1, 1999). All operators were fully gowned, and relevant tools (tubes, needles, dispensers, bottles) sterilized by autoclaving or with ethanol. The cleanliness of the high-purity water used to dilute the base suspensions was confirmed, and the tubes of the dispenser system were rinsed with the suspension to be filled. Apart from that, the ampoules were filled following the procedure described for NanoChOp-03.

### Methods

#### Particle size and zeta potential techniques

Several standardized analytical techniques were used to compare the suspensions with the agreed target properties. This section describes the main measurands and defines the corresponding symbols. Instrument data are provided in the Supplementary Information (3), Table S1.

DLS estimates equivalent spherical hydrodynamic diameters of nanoparticles in a suspension based on the rate of their diffusion due to Brownian motion. The cumulants method for DLS data analysis was used to obtain an average diameter (*d*_DLS, cum_), which is scattered-light intensity-weighted and therefore strongly biased toward higher size values in the case of a non-monodisperse sample (ISO 22412, 2008). The cumulants method also produces a polydispersity index, but this value is not equivalent to the index defined in Section Sourcing and Processing of Suitable Nanoparticle Materials. The NNLS method for DLS data analysis provided particle size *distributions*, and for each identified peak in the distribution a mean value (*d*_DLS, NNLS_). Different *d*_DLS, NNLS_ values are obtained for different weighting methods. Where we report specific particle size data, the weighting basis will be indicated with an additional subscript (_nb_ for particle number, _v_ for volume, _m_ for mass, and _i_ for (different types of signal) intensity).

CLS methods derive equivalent spherical Stokes diameters from particle sedimentation rates. The sedimentation leads to a size fractionation of the particles, and therefore CLS can resolve multiple particle size modes in a sample. The line-start type of disc centrifuge CLS (ISO 13318-2, 2007) was used to provide values of the modal Stokes diameter, *d*_CLS_. This type of CLS, also known as “differential centrifugal sedimentation” (DCS), has been used for the evaluation of the thickness of layers or shells on the surface of nanoparticles (Bell et al., 2013; Krpetic et al., 2013; Kelly et al., 2015).

SAXS is a method which measures the angular distribution of an X-ray beam scattered by suspended particles under small angles in a forward direction. The scattering contrast is caused by electron density differences in the sample. For particles with a sufficiently narrow size distribution, periodic intensity oscillations are observed, the frequency of which can be directly related to the mean equivalent spherical particle diameter, *d*_SAXS_ (Gleber et al., 2010; ISO 17867, 2015).

Electrophoretic light scattering (ELS) deduces zeta potential values (ζ_ELS_) from the electrophoretic mobility of suspended nanoparticles (ISO 13099-2, 2012). Zeta potential was measured because it is related to the surface charge of the nanoparticles, and therefore is an indicator of the stability of electrostatic-based colloidal suspensions.

#### Infrared spectrometry

To investigate the bonds between silica particles and amino-groups, ATR-FTIR spectra were acquired using a Varian 2000 FTIR Scimitar Series (Varian, Inc., USA) spectrometer equipped with a liquid nitrogen cooled mercury cadmium telluride (MCT) detector and fitted with a “Golden Gate” single reflection diamond ATR accessory (Specac Ltd., UK). Five microliters of sample was spread on top of the ATR-element and spectra were taken as dry film after gentle evaporation of the solvent. In general 128 scans were summed up at a nominal resolution of 4 cm^−1^.

#### Microbiology tests

The microbiological load of the nanoparticle suspensions was examined using plating on nutrient agar. Samples were tested in triplicate by spreading 100 μL of the suspensions onto a nutrient agar plate followed by incubation at (34 ± 1)°C for 36 h. Positive control (*E. coli* NCTC 12241, 0.1 CFU/μL, Bioball batch B341) and negative control (sterile water) samples were tested in the same sequence. Colony forming units were counted using visual inspection.

## Results

### Polydispersity

**Figure 2:**
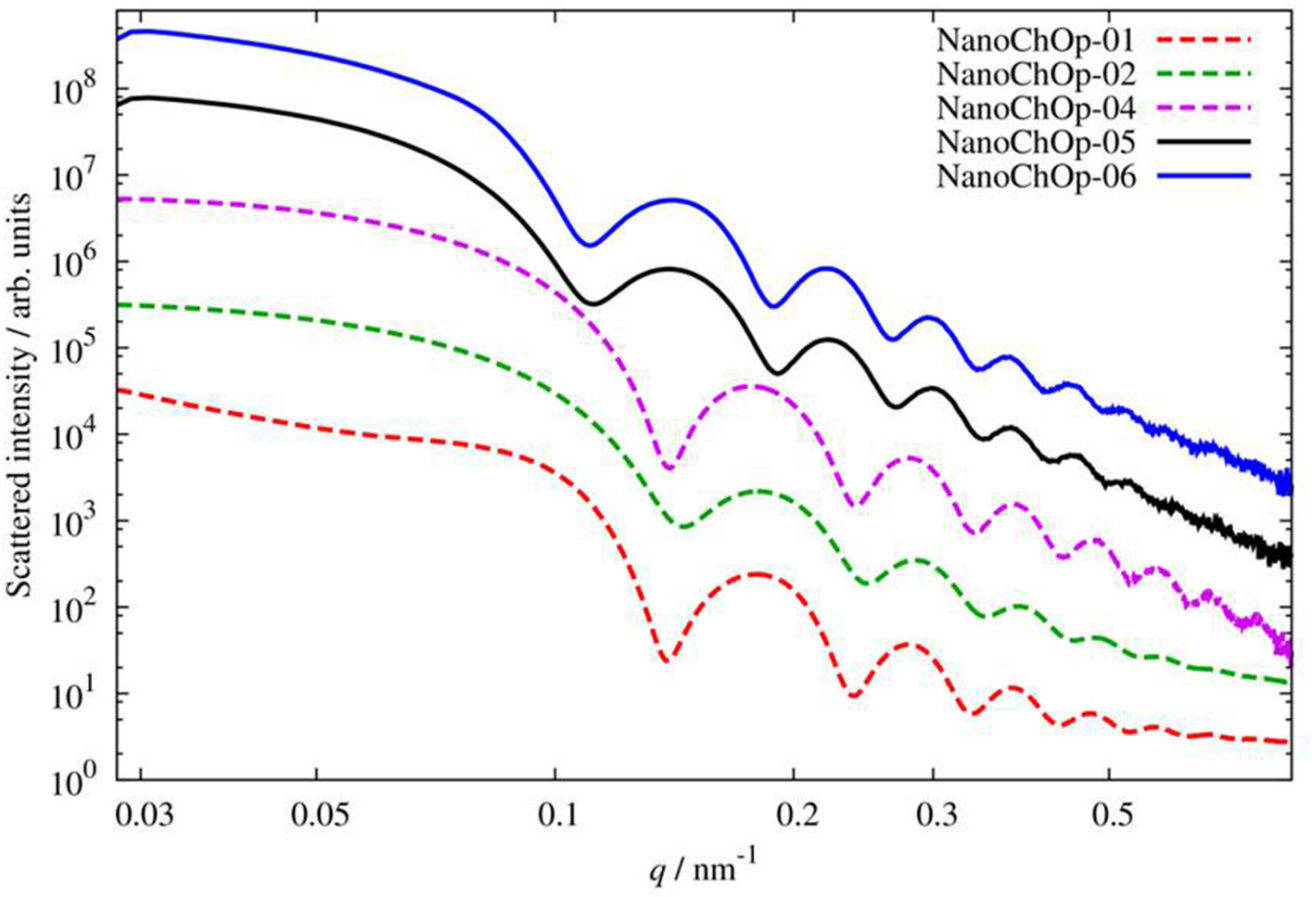
**Scattered X-ray intensity as function of the momentum transfer ***q*** (which in this range is proportional to the scattering angle) for the 5 NanoChOp silica suspensions**.

SAXS measurements revealed that the 5 silica materials met the key requirement of a polydispersity <0.25 (NanoChOp-01: 0.10, NanoChOp-02: 0.15, NanoChOp-04: 0.11, NanoChOp-05: 0.13, NanoChOp-06: 0.13). This was also deduced from the oscillating scattering intensities at higher scattering angles (Figure 2), which facilitate the reliable measurement of *d*_SAXS_. The multi-layered core/shell/shell structure of the NanoChOp-03 QDs did not allow the same straightforward SAXS data analysis.

The NanoChOp-03 base material was affected by agglomeration and contained larger particles that were visible in the ampoules. As a result, DLS data depended strongly on the scattering angle used (Berne and Pecora, 2000). CLS results showed a peak near *d*_CLS, i_ = 14 nm, but the interpretation was inconclusive since this value is close to the lower quantification limit of the method. Filtration was tried (using regenerated cellulose syringe filters with nominal pore sizes of 0.1 and 0.2 μm) as well as centrifugation (60 s at 9300 × g) to remove larger agglomerates. Particle recovery (assessed with photometry) and DLS data suggest that the 0.2 μm pore size filters removed part of the larger agglomerates [see also Supplementary Information (4), Table S2], but not enough to meet the polydispersity target value.

The polydispersities of the NanoChOp-05 material and of its aminated version, NanoChOp-06, measured with CLS, DLS and SAXS, agreed with the preset polydispersity criterion. While only one clear peak was observed in the *d*_CLS, i_ distributions of NanoChOp-05 and -06, near 90 nm, a number of smaller silica particles appear in TEM image (Figure 1) of the base material. CLS measurements performed down to a sufficiently low size range confirm the presence of these particles in the particle number-based *d*_CLS, nb_ distribution (Supplementary Information (6), Figure S4).

### Effective surface functionalization

#### Aminated silica for fluorophore labeling

A key requirement for the aminated silica (NanoChOp-06) was its ability to be fluorescently labeled. This requires that (1) a sufficient number of amine groups is present on the surface of the silica particles, and (2) that they are covalently bonded to the particle surface to serve as strong anchors for the fluorophore groups later.

The presence of amino-groups on the as-aminated silica particle surface is indicated by a pronounced change of the zeta potential of the particles from about −50 mV (NanoChOp-05) to values around 40 mV (immediately after amination and confirmed 1 week later after transport between 2 project partners). After ampouling the aminated silica, to obtain the final NanoChOp-06 material, the zeta potential was lower, but still positive (about 10 mV at pH 3). These positive zeta potential values indicate that there are protonated amino groups on the silica surface. It was noted during an acid-base titration that the NanoChOp-06 aminated silica showed an isoelectric point below pH 4. This is usual for non-surface-modified silica (Metin et al., 2011) and indicates that the particles are not fully aminated, but that was not required for the foreseen fluorophore labeling. New methods to assess the actual amount of amino-groups accessible for dye labeling are currently being developed with the help of the as-aminated and the ampouled NanoChOp aminated silicas.

**Figure 3:**
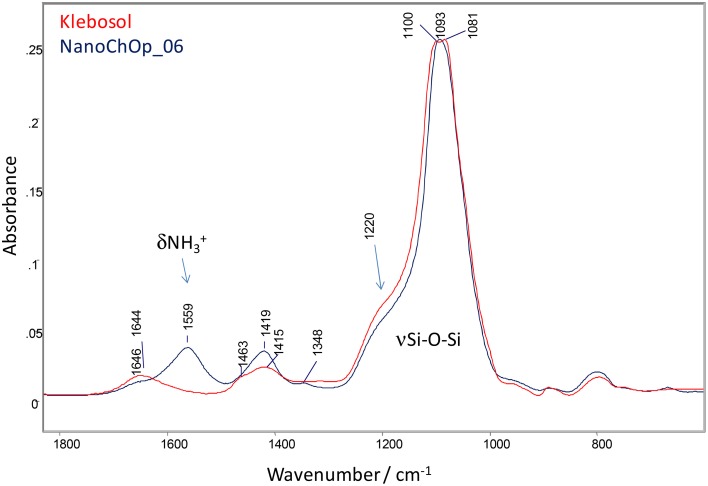
**ATR-FTIR spectral comparison of native silica (Klebosol) and amino functionalized silica (NanoChOp-06) in the aminopropyl deformation and Si-O-Si stretching region**.

As mentioned, a difference was observed between ζ_ELS_ of the NanoChOp-06 material (around 10 mV, immediately after ampouling) and the as-aminated material (around 40 mV). Furthermore, the stability studies (see Results and Data Evaluation) indicate that the zeta potential value of NanoChOp-06 slowly decreases over time. One might therefore question whether the amino-groups are stable and covalently bound on the silica surface. Evidence for the covalent bonding of the amino-groups is the fact that the zeta potential value of 40 mV was measured after dialysis of the reaction mixture for 4 days. Covalent bonding was also concluded from ATR-FTIR results. This technique revealed that the native Klebosol silica contains some aliphatic groups. After silylation (NanoChOp-06) new bands appear: a band at 1559 cm^−1^ belongs to the deformation mode of ${\text{NH}}_{3}^{+}$ groups, formed on the silica surface and stabilized by the acetate ion counterpart. A small band at 1348 cm^−1^ can be related to the CH_3_ deformation of anchored propyl groups. In the spectrum of the pure APDEMS silylation agent the corresponding –NH bending is located at 1599 cm^−1^. The 40 cm^−1^ shift of this band compared to the same band of the amino-functionalized silica sample indicates the covalent bonding of the silylation reagent to the surface. The covalent binding of the silylating agent onto the silica surface can also be witnessed by changes in the spectral features of the broad Si-O-Si stretching band (from 1200 to 900 cm^−1^). The shoulder around 1220 cm^−1^ correlates with the Si-O-Si bonding angle (Nagai and Hashimoto, 2001) and its changes in relative intensity imply the changes in the chemical bonding structure of the silica surface (Pálmai et al., 2013). Comparing the spectral region of the native silica (Klebosol) and that of amino functionalized silica NanoChOp-06 (Figure 3), the changes in band features at 1220 cm^−1^ confirm the covalent binding of the silylation agent onto the silica nanoparticle surface.

Previous studies have shown that the successful fluorophore labeling approach followed in NanoChOp requires adequate amination of the surface (Laux et al., 2012; Felbeck et al., 2015). Standard practice in this approach has been to thoroughly wash (twice with ethanol and twice with water) the materials after labeling, to remove any adsorbed dye from the particles. Successful dye-labeling was demonstrated with absorption and emission spectrometry after these washing steps. Furthermore, the resulting fluorescent particles were successfully applied in the harsh conditions of cell-based confocal fluorescence microscopy, where they exhibited the same emission properties (spectra and relative intensities) as in dispersion. Therefore, covalent bonding of the amine groups is also considered confirmed *a posteriori*, by the results obtained with the fluorescently labeled material.

#### Quantum dots for antibody conjugation

An essential requirement for NanoChOp-03 was its suitability for antibody conjugation for use in a diagnostic assay. Antibody conjugation was performed using an existing protocol described by Hermanson (2008), both with NanoChOp-03 and with another type of commercially available QDs (Nanocrystal 705, Q2206IMP from Invitrogen, Thermo Fisher Scientific, MA, USA). A 1 μM solution of NanoChop-03 particles was activated with sulfo-SMCC cross linker (Thermo-Fisher, Loughborough, UK), at a 20 fold molar excess of antibody, for 60 min at room temperature in phosphate buffered saline (PBS). Excess cross-linker was removed by using a desalting column (NAP-5, from GE Healthcare, Amersham, UK). The resulting particles contained reactive maleimide groups for coupling to the thiol-containing antibody. In parallel, antibody prepared at ~1 g/L was reduced for 30 min with 20 mM dithiothreitol (DTT), followed by desalting (NAP-5) to remove the reducing agent. The conjugation reaction of the maleimide activated particles and thiol-containing antibody both in PBS was performed at room temperature for 60 min. The reaction was quenched by the addition of 10 μL of 10 mM 2-mercaptoethanol. Batches of conjugates were concentrated using Amicom® spin filters (MWCO 50 kDa from EMD Millipore, Watford, UK) at 4000 g. Finally, NanoChop-03 conjugates were purified from unbound antibody using a Superdex 200® (GE healthcare) packed column.

Compared to the Nanocrystal 705 QDs, the recovery (particle concentration) of NanoChOp-03 from the desalting and size exclusion columns used for antibody conjugation and purification was low. In an Interleukin-6 (a chronic wound biomarker, Worsley et al., 2012) assay antibody-conjugated NanoChOp-03 generated a significantly lower binding response to the test line and pre-test line non-specific binding [Figure S1, Supplementary Information (5)]. It was concluded not to pursue further the foreseen bio-assay work with the NanoChOp-03 material, but to revert to the contingency plan, and use carboxylated polystyrene particles instead.

### Biological contamination

#### Detection and diagnosis

Cell culture tests revealed bacterial contamination of NanoChOp-01 and NanoChOp-03. The microbiological load of several base suspensions and ampouled NanoChOp materials was therefore investigated more closely. For NanoChOp-01, viable bacteria counting confirmed the presence of living microorganisms in both the base material and the ampouled material. Optical microscopy indicated that particles visible in the NanoChOp-01 and NanoChOp-04 base materials were not living microorganisms, but their organic nature, deduced from infrared spectrometry, was likely to promote microbial growth. The base material of NanoChOp-02 tested positive for the presence of bacteria, whereas that of NanoChOp-06 tested negative. The ampouled NanoChOp-05 and NanoChOp-06 were free of bacterial or fungal contamination. The latter indicates the success of the preventive measures taken during processing [see NanoChOp-06: Aminated Colloidal Silica and Supplementary Information (2)].

#### Remediation and sterilization

It is not uncommon to add a small amount of biocide to suspensions of nanoparticles to prevent the microbial activity, e.g., in calibrants for PSA instruments. Adding antibiotics after opening the NanoChOp-03 ampoules was effective, but this was not a sustainable option for the NanoChOp materials as they were also developed for use in biological media and cell cultures. Instead, the following approaches were tested.

##### Gamma irradiation

NanoChOp-01 ampoules were submitted to a 6 kGy gamma irradiation, effectively eliminating the viability of the microorganisms present in the suspension. The effect of the irradiation on the polymeric flocs was non-conclusive (some reports indicated more flocculation, some less).

Also some of the NanoChOp-03 ampoules were gamma irradiated, as a test. This effectively eliminated the viability of all microorganisms, but it also resulted in the formation of optically visible, fiber-like particles in the suspension, an increase of *d*_DLS, cum_ from 92 to 108 nm and of the corresponding polydispersity index from 0.27 to 0.32, a decrease in pH from 6 to 5, and a change of ζ_ELS_ from +5 to −5 mV. However, since the gamma irradiation did not affect the particle optical properties, i.e., the absorption and emission spectra and the luminescence decay kinetics, it was decided to submit all remaining NanoChOp-03 samples to gamma irradiation. The fibers formed in the suspension interfered with most PSA methods. Only the *d*_DLS, NNLS, nb_ size distribution was not affected and was used to monitor changes in the size distribution of the NanoChOp-03 QDs.

##### Autoclaving and filtration

Tests on ampoules partly and fully filled with water, respectively, established that the glass ampoules used in NanoChOp could withstand a default autoclaving cycle for liquid materials (max temperature: 120°C, time: 30 min). Autoclaving the NanoChOp-02 silica resulted in slight changes of *d*_DLS, NNLS.i_ and *d*_CLS, i_, but a significantly more negative ζ_ELS_ (-10 mV) and an increased pH (+0.5). For NanoChOp-03, the autoclave cycle tested on a limited number of ampoules, did not result in complete sterilization. Moreover, the autoclaving affected the optical properties of NanoChOp-03. Therefore, autoclaving was not pursued further.

Several attempts were also made to use syringe filters with nominal pore sizes of 0.2 μm. This showed to be an effective means of sterilization for NanoChOp-01 and NanoChOp-03, but it decreased the particle concentration to an undesirable extent (>50%).

### Homogeneity

Homogeneity was studied for the three materials that had not yet been eliminated for further use in the project: NanoChOp-03 (QDs), NanoChOp-05 (colloidal silica), and NanoChOp-06 (aminated colloidal silica). Representative particle size distributions are shown in Supplementary Information (6). Details of the data evaluation and of the measures taken during processing to promote homogeneity are given in Supplementary Information (7).

Homogeneity *between samples* was assessed in terms of particle equivalent diameters and zeta potential (Table 3). Analysis of variance (ANOVA) allowed to separate the between-unit variation (*s*_bb_) from the within-unit variation (s_wb_), in accordance with the process outlined in CRM reports (e.g., Braun et al., 2012; Kestens and Roebben, 2014) and in Supplementary Information (7). The resulting values of the standard uncertainty due to homogeneity (*u*_h_) are shown in Table 3.

**Table 3:** **Summary of the results of the homogeneity studies**.

**Material**	**Measurand**	**Average value**	**Homogeneity standard uncertainty, *u*_h_**
NanoChOp-03	*d*_DLS, NNLS, nb_	31 nm	2 nm
	*d*_DLS, cum_	103.2 nm	2.3 nm
	ζ_ELS_	−1.4 mV	0.6 mV
NanoChOp-05	*d*_DLS, NNLS, i_	94.3 nm	0.9 nm
	*d*_CLS, i_	86.9 nm	0.4 nm
	ζ_ELS_	−48.3 mV	1.8 mV
NanoChOp-06	*d*_DLS, NNLS, i_	89.9 nm	0.3 nm
	*d*_CLS, i_	88.4 nm	0.2 nm
	*d*_SAXS, nb_	81.8 nm	0.02 nm
	ζ_ELS_	9.7 mV	0.8 mV

The *within-unit* homogeneity is closely correlated to the minimum sample intake, which is the lowest amount of sample that is representative for the whole unit. As pointed out earlier, the NanoChOp-05 and -06 ampoules needed to be inverted several times in order to remove a cloud of higher colloidal density, which had formed in all ampoules after filling. The between-unit homogeneity experiments for NanoChOp-06 reported in the previous section were performed using 0.2 mL sample intake for CLS. This sample intake gave acceptable repeatability, demonstrating that the within-unit heterogeneity does not contribute to analytical variation if the samples are ≥0.2 mL. For NanoChOp-03 and NanoChOp-05, similar conclusions can be made: if one inverts the ampoule prior to analysis, then DLS (CLS) results indicate a minimum sample intake of 0.1 mL (0.3 mL) for NanoChOp-03 (NanoChOp-05).

### Stability

The experimental assessment of the stability of a (candidate) RM requires a substantial effort (series of tests under repeatability conditions), as well as time (for the studies simulating exposure over short and longer periods of time). Therefore, the effort was restricted to the three selected materials for which also the homogeneity data were shown.

#### Preventive measures

Stability of test materials can be compromised by several factors, such as exposure to light, oxygen, and higher temperatures during transport and storage. The NanoChOp materials were packed in flame-sealed amber glass ampoules, argon-flushed prior and/or after filling. This eliminates most of the incoming light and reduces largely the exposure to oxygen. For critical dispatches, a temperature monitor and/or non-cooled cool packs as an additional thermal mass to avoid freezing were placed inside the well-insulated parcels. Further effects of time and temperature were investigated in the following dedicated stability studies.

#### Design of an isochronous stability study

**Figure 4:**
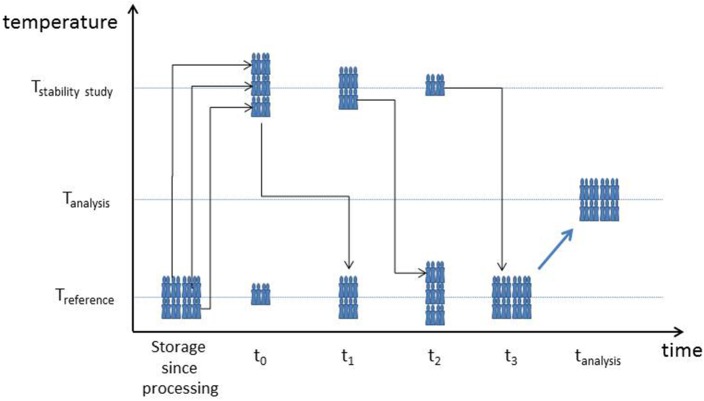
**Schematic presentation of an isochronous stability study, indicating the changes of temperature imposed on a selection of ampoules from the produced batch**. T_stabilitystudy_: temperature for which stability is investigated, T_reference_: temperature for which stability is assured or reasonably assumed, T_analysis_: temperature at which the change of one or more of the material properties is measured. (Note: depending on the test material and test method, the relative position of the three indicated temperatures may change. Also the number of time points can be adapted, as well as the time between time points, e.g., 1 or 2 weeks for stability studies mimicking transport conditions, or 6, 12, or 24 months for studies on stability during long term storage).

The most sensitive studies of the effect of temperature on the stability of a material are performed using an isochronous study design (Lamberty et al., 1998). In such studies, a statistically relevant number of samples is selected and randomly distributed in a test schedule exposing different sets of samples for different periods of time to the selected study temperature(s), as schematically shown in Figure 4. At the end of the study, all samples are measured under repeatability conditions. This facilitates the detection of small differences between samples, here in terms of the same measurands as in the homogeneity study (particle size and zeta potential).

In the short term stability study, exposure times and test temperatures are chosen to mimic transport conditions, Ampoules were stored at temperatures potentially occurring during sample transport (4 and 60°C) for 1, 2, or 4 weeks. In the long term stability study, stability at the preferred storage temperature (18°C) was assessed over a period of 6 or 12 months. An essential element in the design of an isochronous study is the choice of a reference temperature. This is a temperature at which the material is expected to be sufficiently stable or at least more stable than at the temperature(s) for which the stability needs to be investigated. The reference temperature for short-term stability studies for nanoparticle suspensions has been set at 18°C (Braun et al., 2012; Kestens and Roebben, 2014). In the absence of viable microorganisms, these nanoparticle suspensions are typically stable at room temperature when stored or transported for short periods of time. For the long-term stability studies, the question is whether storage at the most convenient temperature (room temperature) is acceptable for longer periods as well. The fall-back temperature was chosen as the reference temperature, namely 4°C. More details of the study set-up are shown in Supplementary Information (8).

#### Results and data evaluation

Table 4 summarizes the main results of the short-term stability studies. For the NanoChOp-03 material, compromised to some extent by the occurrence of optically visible fiber-like particles, the measured *d*_DLS, NNLS, nb_ values did not change significantly during the 4 week period of the studies. Measurements of ζ_ELS_ indicated a statistically significant, but small decrease with time at 60°C (slope = −0.5 mV/week, standard error of the slope = 0.1 mV/week). Therefore, NanoChOp-03 can be shipped without cooling elements, unless prolonged exposure to high temperatures is expected. For NanoChOp-05, the CLS measurements revealed no significant trend. It was concluded that NanoChOp-05 can be shipped under ambient conditions, also based on other prior measurements of the size of these particles. Also for NanoChOp-06, the measured *d*_CLS, i_ values are stable, but the average *d*_DLS, NNLS, i_ values decrease both at 4°C and 60°C. The decrease was small (0.4% for a 2-week transport at 4°C and 0.9% for a 2-week transport at 60°C). Technically more significant was the decrease of ζ_ELS_ in the samples placed at 60°C. Based on these results, it was concluded that NanoChOp-06 can be shipped under ambient conditions. Only if a prolonged exposure to elevated temperatures (>1 week a 60°C) is expected, the samples must be protected with cooling elements to limit the change of ζ_ELS_.

**Table 4:** **Summary of results of the short-term (4 weeks) stability studies**.

**Material**	**Measurand**	**Significant trend**		**Recommended transport temperature**
		**at 4°C**	**at 60°C**	
NanoChOp-03	*d*_DLS, NNLS, nb_	No	No	Ambient (if prolonged exposure to 60°C can be avoided)
	ζ_ELS_	No	Yes	
NanoChOp-05	*d*_CLS, i_	No	No	Ambient
NanoChOp-06	*d*_DLS, NNLS, i_	Small (technically insignificant)	Small (technically insignificant)	Ambient (if prolonged exposure to 60°C can be avoided)
	*d*_CLS, i_	No	No	
	ζ_ELS_	No	Yes	

Table 5 summarizes the main results of the long-term stability studies. No long-term stability test was performed for NanoChOp-03 due to the negative outcome of antibody-conjugation measurements (see Section Quantum Dots for Antibody Conjugation). Nevertheless, the particle size distributions shown in the Supplementary Information (S5) indicate that the *d*_DLS, NNLS, nb_ size distribution was stable for at least 18 months. For NanoChOp-05, all tests indicated a sufficient stability. Based on the measured *d*_DLS, cum_ values an uncertainty contribution was calculated: for a storage period of 36 months the maximum change of this property was predicted to be 1.1% (95% confidence level). A full stability study was not performed for *d*_DLS, NNLS_ or *d*_CLS_ measurements. However, in the period between 2012 and 2015 several DLS and CLS tests were performed and all measured average *d*_CLS, i_ (*d*_DLS, NNLS, i_) values were within the range [87 nm, 89 nm] ([92 nm, 94 nm]), without showing trends in time. Since these *d*_CLS, i_ and *d*_DLS, NNLS, i_ measurements were not performed in an isochronous design, the corresponding expanded uncertainty estimate (2%) is not as small as that obtained for the *d*_DLS, cum_ values. Nevertheless, the NanoChOp-05 material can be stored at room temperature (around 18°C) for at least 3 years without affecting the *d*_DLS, cum_ and *d*_CLS, i_ particle sizes beyond measurement uncertainty.

**Table 5:** **Summary of results of the long-term 18°C stability studies**.

**Material**	**Measurand**	**Significant trend**	**Uncertainty from long-term stability, *U*_lts_(36 months at 18°C, *k* = 2)**	**Recommended storage temperature**
NanoChOp-05	*d*_DLS, cum_	No	1.0 nm	18°C
	*d*_DLS, NNLS, i_	No	2 nm	
	*d*_CLS, i_	No	2 nm	
NanoChOp-06	*d*_DLS, NNLS, i_	Yes	5.3 nm	4°C
	*d*_CLS, i_	No	1.1 nm	
	ζ_ELS_	Yes	2.8 mV	
	*d*_SAXS, nb_	No	0.1 nm	

**Figure 5:**
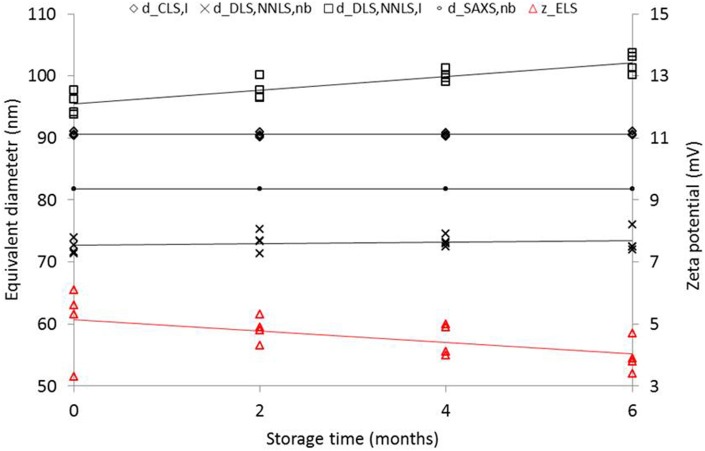
**Change of equivalent diameters and zeta potential of NanoChOp-06 aminated colloidal silica after different periods of storage at 18°C**. Note: the 4 SAXS data points for each storage time cannot be distinguished in this graph due to the excellent repeatability of SAXS.

For NanoChOp-06 *d*_DLS, NNLS, nb_, *d*_CLS_, and *d*_SAXS, nb_ measurements did not detect any change when the material was stored for 6 months at 18°C. On the other hand, *d*_DLS, NNLS, i_ measurements revealed a significant increase over time (Figure 5), which may be related to the simultaneous and statistically significant decrease of ζ_ELS_ from +5.5 mV to +4.0 mV. It was decided to store NanoChOp-06 at 4°C instead of 18°C. Tests performed 18 months after ampouling on samples stored at 4°C indicated unchanged *d*_CLS, i_(near 90 nm) and *d*_DLS, NNLS, nb_ (near 72 nm) values within measurement uncertainty. However, *d*_DLS, NNLS, i_ increased from 90 to 100 nm and ζ_ELS_ decreased from 10 to 5 mV. At this moment, there is no clear explanation for the change of the zeta potential of NanoChOp-06 over time. It is noted that the main change occurred during ampouling (drop from 40 to 10 mV). It is possible that the amino-groups were affected by exposure to oxygen during ampouling. [It is known that some amino groups have to be protected from oxidation, e.g., by cleavable fluorescent labels (Zhang and Chen, 2012)]. Oxidation could have been further reduced by bubbling argon through the base suspension prior to and during ampouling. However, the hypothesis of oxidation is not supported by the results in the next section, on changes of the properties after opening of the ampoules. An alternative explanation may therefore be that acetate ions gradually localize near the particle surface, which reduces the effect of the amino groups on zeta potential and, hence, on agglomeration.

#### Stability after opening

Most RM producers recommend that the content of ampouled nanoparticle suspension CRMs should be used on the day of opening the ampoule (Kestens and Roebben, 2014). However, one of the targets for the NanoChOp materials was a 5-day shelf life after opening (Table 1). Therefore, the stability after opening of the ampoules was investigated.

Analysis of the short-term stability study data for NanoChOp-03 QDs revealed that the results of replicate particle size measurements obtained on a second measurement day differ significantly from the data of the first measurement day. This was accompanied by an increase in the pH (from the original pH 4.7 to pH > 5.5, due to uptake of CO_2_), suggesting that the stability of the samples after opening of the ampoules is limited. Therefore, it was recommended to use the NanoChOp-03 samples at the day of opening.

For NanoChOp-05 silica, the measured particle size values were stable during a 10-day period after opening of ampoules, but both the zeta potential and the pH changed. If the user is only interested in the particle size values, the suspension could be used for 10 days, provided the opened ampoule was kept closed with paraffin film.

Some NanoChOp-06 aminated silica ampoules were opened, flushed with Ar and closed with paraffin film. The particle size and zeta potential measured 14 days later was the same as the values that are measured on freshly opened ampoules. Since flushing with Ar may not be practicable in all circumstances, and because the initial Ar content in the paraffin film-closed ampoules changes (the paraffin film is not leak-tight), it was not recommended to do the Ar flushing. Instead, the paraffin film-closed ampoules were recommended to be used within 5 days after opening.

## Discussion and conclusions

In this section we summarize the data obtained for NanoChOp-03, -05, and -06 to determine for which properties the materials are RMs or RTMs (Properties, Status and Use of the NanoChOp Test Materials). Thereafter (Recommendations for Future Collaborative Research Projects) a number of recommendations are made for future collaborative research projects, some generic, some specific for nanoparticle research.

### Properties, status, and use of the NanoChOp test materials

Tables 6**–8** show the main elements that need to be specified on each RM product information sheet (ISO Guide 30, 2015). Since the property values reported in Tables 6**–8** are not provided with the metrological traceability statement and measurement uncertainty required for certified values, these values can only be considered as first estimates of the true values. However, the tables do indicate for which of their properties the materials meet the requirements for an RTM or an RM, i.e., appropriate homogeneity and stability. Based on reported *u*_h_ and *U*_lts_ values, a user of the materials can decide how much of his experimental variation can come from between-ampoule differences or from the time between different measurements. It is also noted that the *u*_h_ value decreases with $1∕\sqrt{n}$, with *n* the number of ampoules over which results are averaged. Therefore, the *u*_h_ value can be used to calculate the number of ampoules that need to be measured to reduce the corresponding variation below a value chosen by the user of the materials.

**Table 6:** **Properties and status of NanoChOp-03**.

	**NanoChOp-03**			
**General description**	Aminated CdSe/CdS/ZnS QDs, aqueous, nominal concentration 1 mmol/L			
**Specific observations**	Sterilized by gamma irradiation (6.4 kGy)			
	Containing a fraction of fiber-like particles visible to the naked eye			
**Instructions for use**	Minimum sample intake 0.1 mL; store at room temperature; avoid freezing; use contents of an ampoule on the day of opening			
**Equivalent diameters**	**Measured value**	***u*_h_**	***U*_lts_ (36 months)**	**Status**
*d*_DLS, cum_	103 nm	2.3 nm	–	Test material
*d*_DLS, NNLS, nb_	31 nm	2 nm	–	Test material
**OTHER MEASURED PROPERTIES**				
ζ_ELS_	−1.4 mV	0.6 mV	–	Test material
pH	5	–	–	Test material
Absorption maximum (first excitonic peak)	598 nm	–	–	Test material
Emission maximum (FWHM)	612 nm (<30 nm)	–	–	Test material
Photoluminescence quantum yield	0.14	–	–	Test material

Table 6 indicates that the status of NanoChOp-03 QDs does not go beyond that of a test material. The large fiber-like particles prevent it from being used as a reliable RM, also since the long-term stability of the material was not quantitatively evaluated. The material can only be used for the initial development of techniques which are not affected by the larger particles, or after filtration.

Table 7 shows that the homogeneity and stability of the NanoChOp-05 silica are confirmed via *d*_DLS, cum_, *d*_DLS, NNLS, I_, and *d*_CLS, i_ measurements. Therefore, NanoChOp-05 is an RM for these PSA techniques. Although explicit homogeneity and stability data were not obtained with SAXS, the RM status is also assigned to NanoChOp-05 for the SAXS method, based on the combined DLS and CLS information on particle size homogeneity and stability (see Choice of PSA Methods to Investigate Candidate RMs). Because of its excellent stability in terms of particle size, and relying on punctual measurements of ζ_ELS_, the NanoChOp-05 suspension is also considered an RTM for ζ_ELS_, the only additional requirement being that ζ_ELS_ is measured on the day of opening the ampoule.

**Table 7:** **Properties and status of NanoChOp-05**.

	**NanoChOp-05**			
**General description**	Silica nanoparticles, nominal mass fraction 2.5 g/kg			
**Specific observations**	Free of active bacterial contamination			
**Instructions for use**	Remove cloud formation by repeated inversion; minimum sample intake 0.3 mL; store at room temperature; avoid freezing; close opened ampoule with paraffin film; use within 10 days after opening (or on day of opening for measurement of ζ_ELS_)			
**Equivalent diameters**	**Measured value**	***u*_h_**	***U*_lts_ (36 months)**	**Status**
*d*_DLS, cum_	90 nm	–	1.0 nm	RM
*d*_DLS, NNLS, i_	94 nm	0.9 nm	2 nm	RM
*d*_CLS, i_	87 nm	0.4 nm	2 nm	RM
*d*_SAXS, nb_	(81.1 ± 0.8) nm	–	–	RM
**OTHER MEASURED PROPERTIES**				
ζ_ELS_	−48 mV	1.8 mV	–	RTM
pH	8.4	–	–	Test material
Effective particle density	2.0 g/cm^3^	–	–	Test material

Table 8 shows that the homogeneity and stability of the NanoChOp-06 aminated silica are confirmed via *d*_DLS_,_cum_, *d*_CLS, i_, and *d*_SAXS, nb_ measurements. Therefore, NanoChOp-06 is an RM for measurements of these or related equivalent diameters, if it is properly stored (at 4°C) for a period of maximum 36 months. The stability and homogeneity of the measured equivalent diameters are better for methods that are less sensitive to matter attached to or collected on the surface of the particles: the *d*_SAXS, nb_ value is more stable than the *d*_CLS, i_ value, which is more stable than the *d*_*DLS, cum*_ value. However, due to the slowly progressing change of *d*_DLS, NNLS, i_ and ζ_ELS_ detected in the stability studies, NanoChOp-06 is not considered an RM or RTM for measurements of *d*_DLS, NNLS, i_ and ζ_ELS_.

**Table 8:** **Properties and status of NanoChOp-06**.

	**NanoChOp-06**			
**General description**	Aminated silica nanoparticles, nominal mass fraction 2.5 g/kg.			
**Specific observations**	Free of active bacterial contamination			
	Particles visible to the naked eye have formed over time			
**Instructions for use**	Minimum sample intake 0.2 mL; store at 4°C; avoid freezing; close opened ampoule with paraffin film; use within 5 days after opening			
**Equivalent diameters**	**Measured value**	***u*_h_**	***U*_lts_ (36 months)**	**Status**
*d*_DLS, NNLS, i_	89.9 nm	0.3 nm	(5.3 nm when stored at 18°C)	Test material
*d*_CLS, i_	88.4 nm	0.2 nm	(1.1 nm when stored at 18°C)	RM
*d*_SAXS, nb_	(81.8 ± 0.8) nm	0.02 nm	(0.1 nm when stored at 18°C)	RM
**OTHER MEASURED PROPERTIES**				
ζ_ELS_	9.7 mV	0.8 mV	(2.8 mV when stored at 18°C)	Test material
pH	3	–	–	Test material

In the meantime, several publications have been or are being prepared about the work of the NanoChOp project partners using the NanoChOp test materials and the information in Tables 6–8. For example, the NanoChOp-06 aminated silica was used in the development of a new multi-method PSA approach (Bartczak et al., 2015). Although the materials were essentially developed for use within the NanoChOp project, a limited amount of the NanoChOp materials is currently still available for use by third parties, who are willing to share the outcome of the results they obtain.

### Recommendations for future collaborative research projects

#### Choice of PSA methods to investigate candidate RMs

There is a wide range of PSA methods available, each with its stronger and weaker points. For the first time, we have shown how excellent SAXS is to assess quantitatively the homogeneity and stability of the average diameter of the solid core of nanoparticles (Figure 5). The combination of the precision of the SAXS measurements (repeatability better than 0.025%) with the experimental design of the isochronous study resulted in a very small value of the expanded uncertainty on *d*_SAXS, nb_, Since *U*_lts_ <0.1% for a storage period of 36 months, all concerns about the possible dissolution of silica particles in the aqueous suspension in the course of the 3 year project were relieved. The observation also agrees with the expectations for a colloidal silica at pH 3.

Nevertheless, other techniques had to be used to develop a more comprehensive picture of the presence of other particulate fractions. Visual inspection is needed to detect flocculation. DLS is very effective in detecting small numbers of large particles, also if they are of low density (flocs, loose agglomerates). CLS, being a fractionation technique, provided useful information on the presence of smaller signal peaks in the nanorange, e.g., from small aggregates of the main nanoparticle population. It was also shown that for a full understanding and remediation of undesired particulate matter, the mentioned PSA techniques have to be complemented by optical and electron microscopy and chemical analysis techniques.

Different PSA methods provide complementary information. This is why it is not possible to define a single PSA reference method, and why the choice of a PSA method has to be based on the intended use of the measurement results. A practical issue in the production of (C)RMs is the choice of PSA method(s) to perform the required homogeneity and stability studies. Because of the large number of replicate measurements involved, it is desirable to use the homogeneity and stability results obtained with one (or two) methods also in the assessment of homogeneity and stability for other PSA methods. For example, it is possible to use a combination of DLS and CLS data to estimate the stability of a material in terms of SAXS data, because DLS and CLS are sensitive to small changes in particle size distributions, e.g., due to agglomeration (Braun et al., 2012; Kestens and Roebben, 2014). Therefore, it is also recommended not to use the very precise *d*_SAXS_ values to estimate the stability of *d*_DLS_ and *d*_CLS_ values.

#### Characterization of functional groups on nanoparticle surfaces

One of the most challenging aspects of the NanoChOp project was the preparation of particles with a defined surface functionality. The NanoChOp-06 silica proved to carry sufficient amine surface groups to enable fluorophore labeling, but the NanoChOp-03 QDs did not perform as required in the antibody conjugation process needed for their use in an immunoassay. In this respect, it is noted that the methods to assess the number and kind of molecules attached to nanoparticles require further development. Currently available methods analyze the surface of large numbers of nanoparticles collectively. A critical input parameter for the interpretation of these ensemble analysis methods is the nanoparticle number concentration, which cannot be reliably measured to-date except the particle size distribution is very monodisperse. The long-term ambition is to find methods that provide information on the groups present on the surface of individual nanoparticles without having to remove the particles from the application-relevant medium. First results, using markers that attach to functional groups on the nanoparticle surface to make the distribution of the functional groups visible with electron microscopy, have been published recently (Kelly et al., 2015).

#### Microbiological contamination of nanoparticle suspensions

Several of the tested candidate suspensions contained bacteria, in particular the suspensions with organic residues from particle preparation. ‘Gamma irradiation was already shown to be an effective means of sterilizing nano-objects (Fagan et al., 2011). Our results indicate that dosages between 6 kGy and 8 kGy are an effective method to abrogate the viability of microorganisms in ampouled aqueous suspensions of silica(-based) and QD particles. However, in the case of the NanoChOp-03 QDs the gamma irradiation was one of the possible causes of formation of aggregates and agglomerates. It was also shown that the flame-sealed amber-colored ampoules (size 5–10 mL) can mechanically withstand standard autoclaving protocols, but not without affecting the physicochemical properties of the NanoChOp-02 silica suspension. An obvious conclusion is that whenever possible, the microbiological contamination shall be prevented. The processing of the NanoChOp-06 base material showed that this is possible, using standard autoclaving and ethanol decontamination procedures of the applied tools and by ampouling under a mobile clean-cell.

#### Managing the ambitions of a collaborative research project to develop RMs

Comparing Table 1 with Tables 6–8 reveals that the original target properties for the NanoChOp test materials were ambitious. Our findings confirm that the selection of common test materials is a critical step in the design and execution of a collaborative research project. This is particularly the case for research projects that aim to establish and use *reference materials* in their experiments. This paper has illustrated how to verify the required homogeneity and stability of the selected test materials experimentally if they are intended for use as an RM or RTM. In particular, the uncertainty contribution due to between-unit variation, *u*_h_, has to be assessed because a precise assessment of the reproducibility of a method between different laboratories is not possible without knowing the homogeneity of the test material. Also the design and results of long-term stability studies have been shown, as they indicate whether or not data obtained on the selected materials at the start and close to the end of the project can be reliably compared.

It can be argued that the selection of candidate test materials is an ordinary part of the planning phase of any research project. It must be noted however that the amounts of test material in collaborative research projects with multiple partners can be considerable. Moreover, the homogeneity requirements for a RM imply that the materials are best processed as a single batch, often exceeding the typical capacity of a research laboratory. Furthermore, the evaluation of homogeneity and stability of a candidate RM require a considerable number of tests, in particular when the material is to be used across a range of properties and techniques. *De facto*, this is an effort that requires resources that are usually not available prior to the start of a research project. Instead, the suitability of selected materials will only be revealed throughout the course of a research project. Therefore, flexibility will be required in the preparation of common test materials for use in collaborative research projects, both from the provider, who as a rule will face changing or additional material requirements especially in the early stages of the project, and from the test material users, who often will have to face the fact that a material perfectly meeting all requirements is not available. It should, however, always be the intention to minimize this need for flexibility, and for taking decisions based on other than scientific grounds, e.g., for reasons of time or financial constraints. This is why the intended use of the test materials in the project needs to be carefully considered before candidate materials are selected.

In view of the above discussion, it is recommended that the managers of research projects working with common test materials develop periodically updated product information sheets (ISO DGuide 31, 2014) to monitor, during the course of a project, the information gradually becoming available on the homogeneity and stability and other aspects of the selected test materials, such as instructions for use (Tables 6–8). These sheets should collect and merge the essential data from different project partners and clarify the evolving status of a test material (from test material to RTM or RM), taking into account that this status is different depending on the use of the material. The authors hope that further dissemination and implementation of such practices will eventually lead to a broader range of commercially available nanoparticle CRMs, RMs, and RTMs to support collaborative projects, which would result in saving substantial time and financial investment for the individual projects.

### Conflict of interest statement

The authors declare that the research was conducted in the absence of any commercial or financial relationships that could be construed as a potential conflict of interest.
