# Supplementary material for: Reference materials and representative test materials to develop nanoparticle characterization methods: the NanoChOp project case
Source: Front Chem. 2015 Oct 19;3:56. doi: 10.3389/fchem.2015.00056 (PMC4609882; doi:10.3389/fchem.2015.00056)
Supplement: Supplementary file 1 [file DataSheet1.PDF]

Supplementary Material

Reference materials and representative test materials to develop nanoparticle characterization methods: the NanoChOp project case

Gert Roebben, Vikram Kestens, Jean Charoud-Got, Yannic Ramaye, Stephane Mazoua, Nele Meeus, Philippe Corbisier, Marcell Palmai, Judith Mihály, Zoltan Varga, Dorota Bartczak, Julie Davies, Christian Gollwitzer, Michael Krumrey, Daniel Geißler, Ute Resch-Genger, James Noble, Neelam Kumarswami, Caterina Minelli, Aneta Sikora, Heidi Goenaga Infante

*** Correspondence:** Corresponding Author: gert.roebben@ec.europa.eu

1. Supplementary information (1): Sourcing, processing and properties of the test materials NanoChOp-01, -02 and -04

NanoChOp-01: aminated colloidal silica

Initially, a suspension of aminated 'hybrid' silica nanoparticles was selected from a series of candidate silica materials, as it had the lowest polydispersity. The *hybrid* silica nanoparticles contain an organic fraction, resulting in a lower particle density (around 1.3 g/cm^3) than that of common colloidal silica (around 2 g/cm^3). Traces of a surfactant used as a stabilizing agent during particle processing were held responsible for foam formation observed in a first test batch. A second batch had undergone additional purification steps and was used for the production of NanoChOp-01. The supplied suspension was diluted and ampouled as described for NanoChOp-03, following processing parameters summarized in Table 2. It was soon noticed that the ampouled NanoChOp-01 material contained a viable microbiological load. Therefore, ampoules were gamma-irradiated as described for NanoChOp-06.

In the NanoChOp-01 ampoules, a number of flocs were visible. These did not perturb the SAXS or CLS measurements. CLS indicated a main peak in the size distribution near 60 nm, and a small second peak, near 70 nm, probably corresponding with dimer particles. DLS data obtained at a forward scattering angle (13°) were affected by the visible flocs, as they showed a main peak in the $d_{DLS,NNLS,i}$ size distribution near 250 nm, which disappeared after filtering the sample, leaving only the peak near 100 nm.

NanoChOp-02: non-functionalised colloidal silica

Despite several offers from commercial producers and research laboratories, it was not possible to obtain a well-dispersed and stable suspension of TiO_2 particles in the desired size range (near 20 nm) with a sufficiently low polydispersity. The material that came closest was an anatase TiO_2 prepared through solvothermal synthesis, developed for use in photocatalysis (Parnassos, NAMA41N, Colorobbia, Sovigliana, IT). Unfortunately, the size distributions measured with DLS and transmission electron microscopy (TEM) showed the presence of groups of particles with different average sizes (around 5 nm, around 20 nm and around 100 nm), and the suspension showed signs of flocculation. Sonication, dilution, pH modification, centrifugation and filtration were attempted to improve the size distribution, but only with moderate success or at the expense of losing nanoparticles. When discussing in detail the requirements listed in Table 1 with the candidate suppliers, they confirmed that the production of a stable TiO_2 suspension meeting these requirements of low polydispersity, neutral pH and absence of surfactants (Table 1) would be challenging. This can also be deduced from scientific literature (e.g. Perez Holmberg et al., 2013), which shows that bare TiO_2 nanoparticles have a point of zero charge near pH 7. It was therefore decided to follow the foreseen contingency plan and to change target material, replacing the TiO_2 material for a non-functionalized silica.

NanoChOp-02 was made from colloidal silica supplied by Grace (Columbia, MD, USA). The processing approach followed was similar to that of NanoChOp-06 (see also Table 2). DLS and CLS confirmed the monomodal and sufficiently narrow size distribution observed with SAXS.

NanoChOp-04: carboxylated colloidal silica

As the preparation of a aminated silica was facing problems that threatened to delay the project, it was decided to also develop an alternative material, i.e. a carboxylated colloidal hybrid silica (NanoChOp-04). NanoChOp-04 was based on a carboxylated version of the silica nanoparticles used for NanoChOp-01 (Table 2). The base material was an opaque white suspension.

Similar to NanoChOp-01, the polydispersity of the NanoChOp-04 base material was acceptable if judged only on the basis of CLS and SAXS data. However, the DLS data showed a multimodal size distribution, indicating the presence of larger agglomerates and particles, as confirmed with optical microscopy. Further purification of NanoChOp-04 was explored, revealing that centrifugation (10 min at 2000 x g) could remove large precipitates, and that filtration of the supernatant (with a

PD-10 column, GE Healthcare, containing a size exclusion (Sephadex G-25) gel with fractionation range of 1-5 kDa) removed a contamination by molecules smaller than the silica particles. DLS data indicated that the resulting purified sample was monodisperse with an average $d_{\mathrm{DLS,NNLS,i}}$ near 100 nm. Nevertheless, TEM showed the presence of some remaining contamination with lower electron density but in the same size range as the particles. Because of lack of purity (discussed in Section 3.1) the further preparation of NanoChOp-04 was stopped.

2. Supplementary information (2): Measures to avoid contamination during ampouling

When interference of bacteria with downstream toxicological studies was reported, some precautions were taken to prevent contamination in the processing of other NanoChOp suspensions (see 2.2.3).

Possible contamination of the nanoparticle suspensions with foreign particles from the glass ampoules was studied in more detail. In their as-received state, the glass ampoules, prepared from long glass tubes, are closed. Prior to filling the ampoules are opened with a flame and after filling the ampoules are flame-sealed again. The question was raised whether the glass ampoules contained small particles that could interfere with size distribution measurements on the nanoparticle suspensions contained in the ampoules. As a precautionary measure, several batches were produced with ampoules that had been previously opened and cleaned and placed in an upright position in an oven to dry, prior to being put on the ampouling machine. This cleaning process was also questioned, as it could affect the sterility of the ampoules, which is ensured by the high-temperature glass processing, at least until the ampoules are opened. To control the effectiveness of the cleaning process, cleaned and non-cleaned ampoules were filled with ultrapure (reverse osmosis purified and sanitized) water and flame-sealed. Water taken from these ampoules was subsequently analyzed with DLS. The resulting scattered light count rates were compared to the count rates measured for water taken from cleaned ampoules. Closely matching values indicated that the ampoules were clean, or, at least, that the cleaning process does not remove particles from the as-received ampoules. Thus, for the type of ampoules tested, a cleaning process is not required.

3. Supplementary information (3): Analytical instruments

Table S1: Details of the analytical instruments and default test parameters used

method	Instrument type	Test parameters
**SAXS**	SAXS set-up at the PTB FCM beamline at BESSY II	Photon energies in the range from 6 keV to 10 keV, sample-detector distances in the range between 2.3 m and 4.5 m, detector pixel size 172 µm
**CLS**	Disc centrifuge DC20000, CPS Instruments (USA)	Line-start CLS with turbidimetric detection (acc. ISO 13318-2, calibrated with PVC standards from CPS Instruments), 20000 rpm, sucrose gradient (20 g/kg to 80 g/kg), 0.1 mL sample volume
**DLS**	Zetasizer Nano ZS, Malvern (UK)	He-Ne laser (4 mW, 633 nm), back-scattering (173°), quartz microcuvette, 0.1 mL sample volume
**ELS**	Zetasizer Nano ZS, Malvern (UK)	He-Ne laser (4 mW, 633 nm), forward-scattering (13°), polycarbonate folded capillary cell with Au-plated Be/Cu electrodes, 0.8 mL sample volume, Smoluchowski model

4. Supplementary information (4): effect of filtration and centrifugation on the polydispersity of NanoChOp-03 QDs

Table S2: NanoChOp-03 particle recovery estimates derived from UV-vis spectrometry (absorbance at the first excitonic peak, measured using a 1 cm path length cuvette and with the solvent blank subtracted) and DLS data ($d_{DLS,cum}$ and $d_{DLS,NNLS,i}$ with their respective polydispersity indices) before and after processing to remove agglomerates (using filtration (0.22 μm) or centrifugation for 1 minute at 10,000 x g).

Sample	Recovery (%)	DLS (cumulants method)		DLS (NNLS method)	
		$d_{DLS,cum}$ (nm)	Polydispersity index (nm/nm)	$d_{DLS,NNLS,I}$ (nm)	Polydispersity index (nm/nm)
**NanoChOp-03**	100	108.3 ± 1.5	0.35	33 ± 7	0.49
**NanoChOp-03, after filtration**	50	70.4 ± 2.3	0.30	33 ± 4	0.49
**NanoChOp-03, centrifuged**	80	96.9 ± 1.0	0.27	37 ± 3	0.49

5. Supplementary information (5): lateral flow strip tests after conjugation of NanoChOp-03 QDs

NanoChOp-03 and Nanocrystal 705 conjugates were run on a lateral flow strip (50 µl running buffer (50 µl borate, 3% BSA, 0.05% tween 20, pH 7.4) mixed with 2 µl of each conjugates) to confirm the activity on control line only. Lateral flow strips (Fig. S1) were analyzed with a fluorescence reader using a band-pass filter (700-710AG40 and FB600-40).

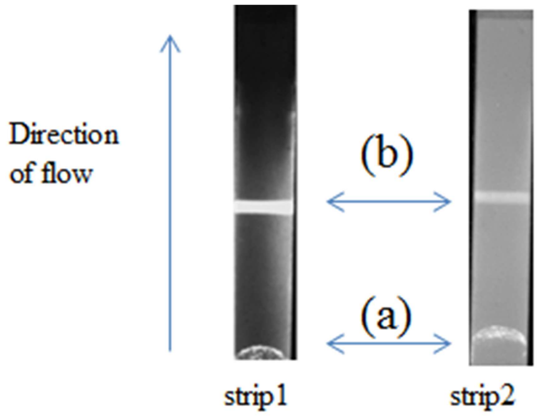

Figure S1: Visual comparison of NanoChOp-03 (strip 2) to Nanocrystal 705 (strip 1) showing additional pre-test crashing of antibody-QD at the beginning of the strip (a), resulting in less specific binding to the control line (b).

6. Supplementary information (6): Particle size distributions of the selected test materials

The following graphs show particle size distributions for NanoChOp-03, NanoChOp-05 and NanoChOp-06. Of the methods listed in section 2.3.1, the most detailed particle size distributions are obtained with CLS. Only for NanoChOp-03 $d_{DLS,NNLS}$ results are shown because the size of the NanoChOp-03 QD particles is close to the lower limit of quantification of the CLS method.

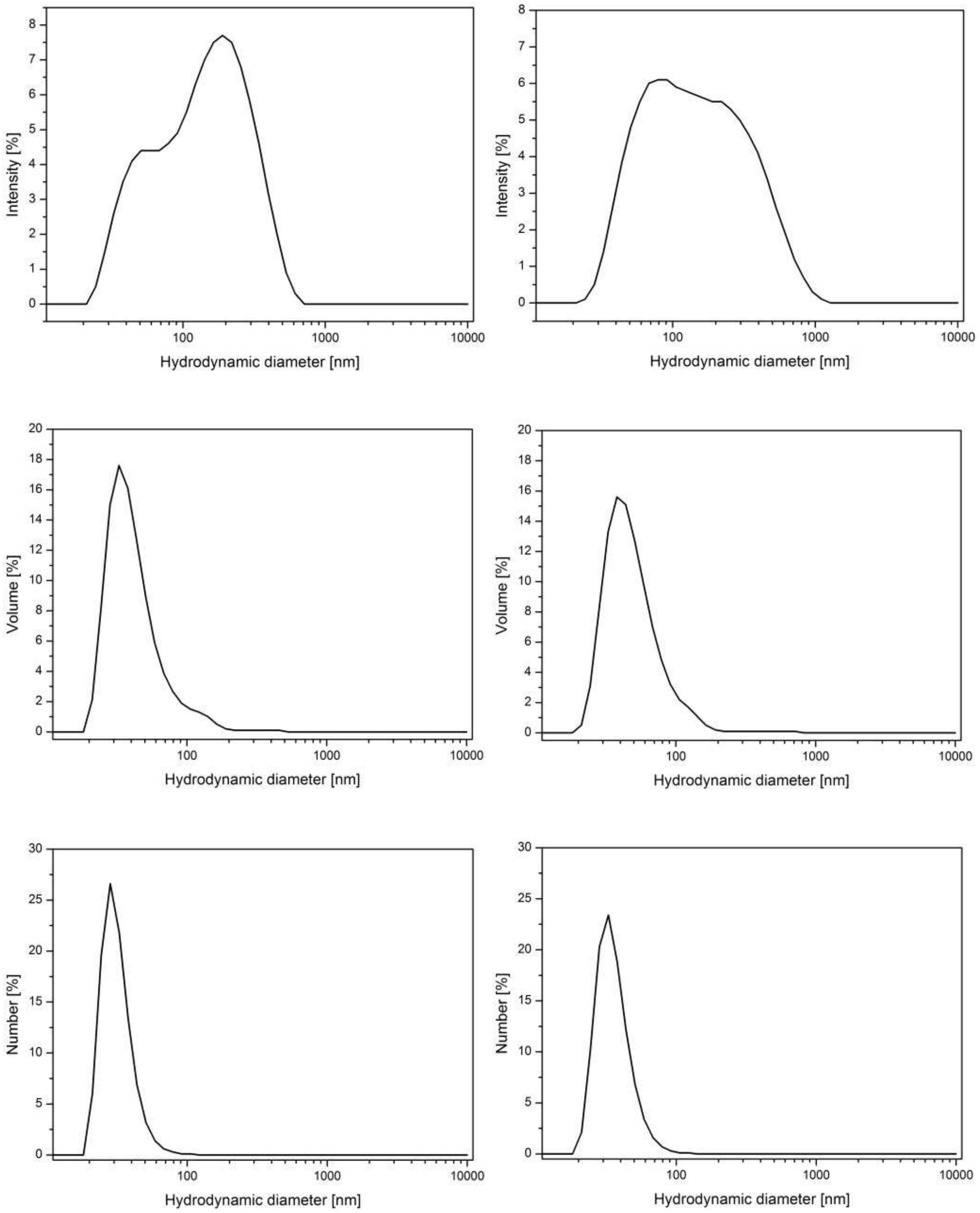

Figure S2: Particle size distributions of NanoChOp-03 QDs ($d_{DLS,NNLS,i}$, $d_{DLS,NNLS,v}$ and $d_{DLS,NNLS,nb}$) measured 2 months (left) and 18 months (right) after gamma irradiation.

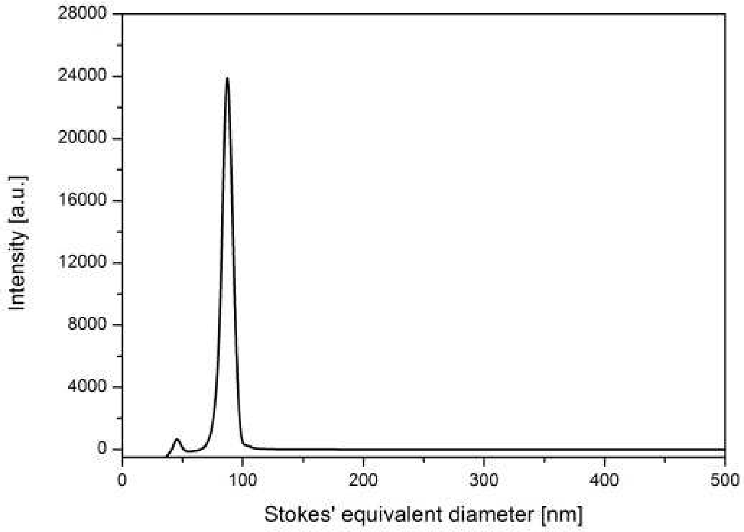

Figure S3: Particle size distribution of NanoChOp-05 silica (d_{CLS}), stable throughout the project.

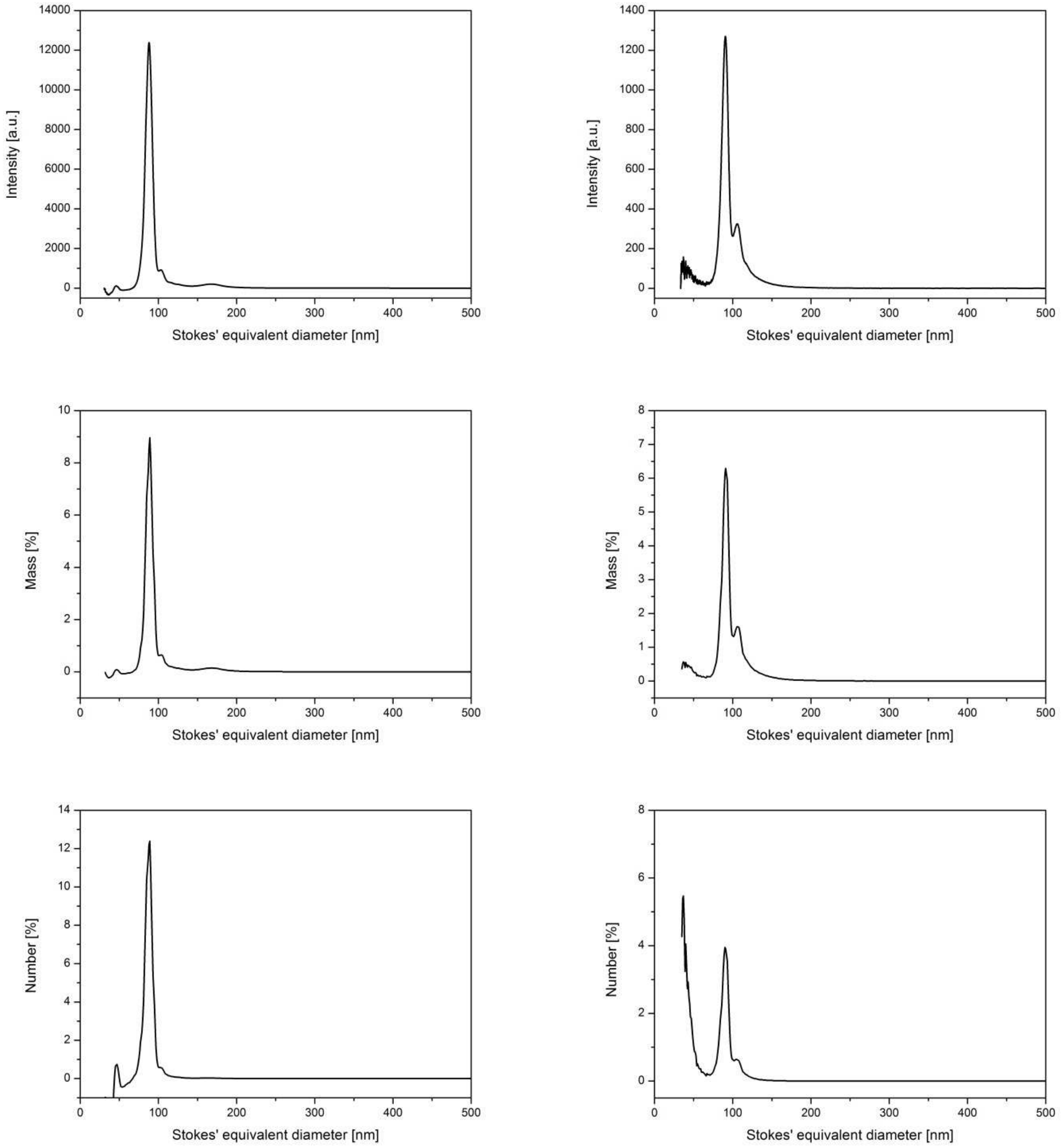

Figure S4: Particle size distributions of NanoChOp-06 aminated silica ($d_{CLS,i}$, $d_{CLS,m}$ and $d_{CLS,nb}$), measured 1 month (left) and 18 months (right) after ampouling.

7. Supplementary information (7): Details of the homogeneity studies

Homogenization during processing

All materials were produced by filling the ampoules from a single vessel in a single run. Homogeneity between ampoules can be affected by processes of selective adsorption or sedimentation, especially of larger (contaminant) particles, prior to and during the filling of the ampoules. Several previously tested approaches were followed to ensure that the content of all ampoules was similar in particle concentration and size distribution. The approach was chosen based on the presence of larger particles, the volume of the available material, and the time needed to perform the ampouling.

The time needed to fill the 228 NanoChOp-03 ampoules was limited (< 1 h). In normal conditions, a magnetic stirrer is used to promote homogeneity of the suspension throughout the glass bottle from which the ampoules are filled. However, as this would result in loss of some of the precious QDs, and to avoid possible interaction between the magnetic stirrer and the QDs, the glass bottle was simply shaken gently before and halfway through the ampouling sequence.

The 400 NanoChOp-05 samples are part of a larger batch of 1871 ampoules, for which the required ampouling time was substantial (> 4 h). Therefore, the ampoules were filled by manual dispensing from a glass bottle in which a magnetic stirrer was continuously operating.

The number of NanoChOp-06 ampoules to be filled (536) was intermediate between the NanoChOp-03 and NanoChOp-05 cases. It was decided not to stir the suspension during filling. Instead, the plastic bottle containing the material before ampouling had rested the night before ampouling so as to allow settling of potential larger particles. Immediately prior to ampouling, the suspension was pumped from the plastic bottle into a glass bottle, except the bottom part with potential sedimentation residues.

Quantitative analysis of between-unit variation of particle sizes and zeta-potential

The number of units to be randomly selected from a batch to test its homogeneity is approximately the cubic root of the number of units (for NanoChOp-03: 6 of 184, NanoChOp-05: 8 of 400, NanoChOp-06: 8 of 536) (ISO Guide 35:2006). Independent aliquots were taken from each selected unit and analysed in duplicate or triplicate. All measurements were performed under repeatability conditions and in a randomised manner to be able to separate a potential analytical drift from a potential trend in the filling sequence. No trends in the filling sequences were detected (Table S3).

All data followed a normal and unimodal distribution. Data were checked and scrutinized for single and double outliers by applying the Grubbs' test at a confidence level of 99 %. Table S3 summarizes the experimental set up of the homogeneity studies and of the trend and outlier analysis of the raw data.

Table S3. Summary of trend and outlier analysis of data from the homogeneity studies

Material	Measurand	Aliquots per unit / Replicates per aliquot	Analytical drift	Outlying mean
NanoChOp-03	$d_{DLS,cum}$	1 / 3	No	No
	$d_{DLS,NNLS,nb}$	1 / 3	No	No
	ζ_{ELS}	1 / 3	No	No
NanoChOp-05	$d_{DLS,NNLS}$	2 / 3	No	1
	d_{CLS}	2 / 1	No	No
	ζ_{ELS}	2 / 3	No	1
NanoChOp-06	$d_{DLS,NNLS}$	2 / 3	No	No
	d_{CLS}	2 / 3	< 0.5 nm / 10 samples	No
	d_{SAXS}	½	No	No
	ζ_{ELS}	2 / 3	No	No

Table S4 presents the results of several homogeneity parameters deduced from the raw data of the homogeneity study.

Table S4. Summary of technical/statistical analysis of data from the homogeneity studies

**Material**	**Measurand**	**Average value**	s_{wb} 1)	s_{bb} 2)	u^*_{bb} 3)	u_{rec} 4)	u_h 5)
NanoChOp-03	$d_{DLS,NNLS,nb}$	31 nm	-	2 nm	-	-	2 nm
	$d_{DLS,cum}$	103.2 nm	-	2.3 nm	-	-	2.3 nm
	ζ_{ELS}	-1.4 mV	-	0.6 mV	-	-	0.6 mV
NanoChOp-05	$d_{DLS,NNLS,i}$	94.3 nm	0.4 nm	(outlier)	0.2 nm	0.9 nm	0.9 nm
	$d_{CLS,i}$	86.9 nm	0.2 nm	0.4 nm	0.1 nm	-	0.4 nm
	ζ_{ELS}	-48.3 mV	2.0 mV	(outlier)	1.0 mV	1.8 mV	1.8 mV
NanoChOp-06	$d_{DLS,NNLS,i}$	89.9 nm	0.6 nm	0.2 nm	0.3 nm	-	0.3 nm
	$d_{CLS,i}$	88.4 nm	0.4 nm	($MS_{between} < MS_{within}$)	0.2 nm	-	0.2 nm
	$d_{SAXS,nb}$	81.8 nm	0.02 nm	($MS_{between} < MS_{within}$)	0.01 nm	-	0.02 nm
	ζ_{ELS}	9.7 mV	0.7 mV	0.8 mV	0.3 mV	-	0.8 mV

[1] Variation (standard deviation) within ampoules, [2] Variation (standard deviation) between ampoules, [3] Maximum variation between ampoules, hidden by analytical imprecision, [4] Between-ampoule heterogeneity estimated as width of a rectangular probability distribution, [5] Homogeneity standard uncertainty

It is noted that s_{bb} and s_{wb} are only estimates of the true standard deviations and are therefore subject to random fluctuations. Therefore, the mean square between groups (MS_{between}) can be smaller than the mean squares within groups (MS_{within}), resulting in negative arguments under the square root used for the estimation of the between-unit variation, whereas the true variation cannot be lower than zero. If s_{bb} cannot be calculated as $MS_{\text{within}} < MS_{\text{between}}$, then u^{*}_{bb}, the maximum inhomogeneity that could be hidden by method repeatability, was calculated as described by Linsinger et al. (Linsinger et al., 2001). The u^{*}_{bb} is comparable to the limit of detection of an analytical method and yields the maximum inhomogeneity that might be undetected by the given study setup. In the case of one outlying unit mean, the between-unit heterogeneity is modeled as a rectangular distribution limited by the largest outlying unit average. The rectangular relative standard uncertainty of homogeneity is estimated as $u_{rec} = \frac{|outlier - \bar{y}|}{\sqrt{3}\,\bar{y}}$ where $\bar{y}$ is the average of all results of the homogeneity study. The larger value of s_{bb}, u_{rec} or u^{*}_{bb} was used as the homogeneity uncertainty, u_{h} (Table 4).

8. Supplementary information (8): Details of the stability studies

Tables S5 and S6 provide additional details of the set-up of the short- and long-term stability studies.

Table S5. Summary of results of the short-term (4 weeks) stability studies

Material	Method	Samples / time point	Aliquots per unit
**NanoChOp-03**	$d_{DLS,NNLS,nb}$	2	2
	ζ_{ELS}	2	1
**NanoChOp-05**	d_{CLS}	4	2
**NanoChOp-06**	$d_{DLS,NNLS}$	2	2
	d_{CLS}	2	2
	ζ_{ELS}	2	2

Table S6. Summary of results of the long-term 18°C stability studies

Material	Length storage test	Method	Samples / time point	Aliquots per unit
**NanoChOp-05**	12 months	$d_{DLS,cum}$	8	2
**NanoChOp-06**	6 months	$d_{DLS,NNLS}$	2	2
	6 months	d_{CLS}	2	2
	6 months	ζ_{ELS}	2	2
	6 months	d_{SAXS}	2	1
